# A novel platform for heterologous gene expression in *Trichoderma reesei* (Teleomorph *Hypocrea jecorina*)

**DOI:** 10.1186/1475-2859-13-33

**Published:** 2014-03-06

**Authors:** Mikael Skaanning Jørgensen, Dominique Aubert Skovlund, Pia Francke Johannesen, Uffe H Mortensen

**Affiliations:** 1 Department of Systems Biology, Center for Microbial Biotechnology, Technical University of Denmark, Building 223, DK-2800 Lyngby, Denmark; 2 Novozymes A/S, Kroghoejsvej 36, DK-2880 Bagsvaerd, Denmark

**Keywords:** Expression, *pyr2*, *Trichoderma reesei*, Defined integration

## Abstract

**Background:**

The industrially applied filamentous fungus *Trichoderma reesei* has received substantial interest due to its highly efficient synthesis apparatus of cellulytic enzymes. However, the production of heterologous enzymes in *T. reesei* still remains low mainly due to lack of tools for genetic engineering.

**Results:**

In this study we present new genetic tools for *T. reesei* to further expand its use in industrial production. We have developed an expression platform where genes are inserted into a versatile expression vector via highly efficient uracil-excision cloning and subsequently inserted into a defined position in the *T. reesei* genome ensuring that enzyme production from different transformants can be directly compared. The *ade2* locus was selected as integration site since *ade2* mutants develop red pigment that facilitates easy and rapid detection of correctly targeted transformants. In addition, our system includes a *tku70* disruption to increase gene targeting efficiency and a new bidirectional marker, *pyr2*, for iterative gene targeting. The dual selection system, color and prototrophism, ensures that correct transformants containing the desired gene inserted into the defined expression site can be selected with an efficiency approaching 100%.

**Conclusions:**

The new genetic tools we have developed are suitable for high-throughput integration of genes into the genome of *T. reesei* and can easily be combined with techniques for generation of defined mutants. Moreover, the usability of the novel expression system with *ade2* as integration site was confirmed by expression of a *Thermomyces lanuginosus* lipase.

## Background

*Trichoderma reesei* is a key workhorse for commercial scale production of different enzymes used by the bioethanol, pulp, paper, and textile processing industries. This status is a consequence of its unique capability of producing and secreting large amount of enzymes, its amenability to large-scale fermentation as well as its long history of safe use in industrial enzyme production. Importantly, production of several *T. reesei* enzymes has obtained the generally recognized as safe (GRAS) status by the U.S. Food and Drug Administration (FDA). Moreover, *T. reesei* serves as a model organism for the regulation of expression and biochemistry of (hemi-)cellulose degradation enzymes and pathways [1,2]. There is an increasing demand for these enzymes since they are employed for the saccharification of cellulosic plant biomass to simple sugars for biofuel production. As enzymes constitute an important cost in the production of bioethanol, large research efforts as well as large government funding have aimed to continuously improve *T. reesei* as an enzyme production host. This process will be greatly facilitated by the fact that its genome has recently been sequenced setting the stage for strain development by directed genetic engineering [3].

Unfortunately, the highly efficient protein synthesis machineries of *T. reesei*, which enable yields of homologous proteins in excess of 100 g/l [4], has so far not prevailed for synthesis of heterologous proteins and yields remain low [5]. Methods and tools to improve synthesis of heterologous proteins in this fungus are therefore highly desirable.

One bottle neck towards this goal has been the low efficiency of gene targeting in *T. reesei*, but like in a number of other fungi this problem has been dramatically reduced by deleting a gene involved in Non-Homologous End-Joining (NHEJ). In such strains the frequency of successful integration by gene targeting was first reported to increase to >95% from the 5-10% obtained with wild-type strains [6]. However, there is still room for improvement as a recent study in *T. reesei* has demonstrated that the efficiency of homologous integration in NHEJ deficient strains can be highly site specific and may vary from 33 to 100% depending on insertion site [7].

Before *T. reesei* can be routinely used for heterologous protein production, it is necessary to gain insights into e.g. the influence of promoter sequences, effects of codon optimized synthetic genes, or expression rates of selected orthologous genes. This type of analysis requires construction of large numbers of strains where the genes to be compared are expressed from a defined locus in isogenic strains to allow for proper comparisons. Moreover, such analysis may often include experiments that require multiple rounds of genetic engineering in the same strain. Consequently, the need for molecular tools that allow easy genetic modifications of *T. reesei* for industrial strain development are urgent. We have therefore developed a new expression platform in *T. reesei* that facilitates heterologous gene expression from a defined locus with an improved gene-targeting throughput. Our expression platform is composed of four parts: 1) a versatile integration plasmid for gene expression containing a USER (Uracil-Specific Excision Reagent) cassette for highly efficient ligase-free USER cloning, 2) a bidirectional marker *pyr2*, encoding orotate phosphoribosyl transferase, that allows for iterative gene targeting, 3) a *tku70* gene disruption strain for efficient gene targeting, and 4) a color marker that facilitates identification of correctly targeted strains, even in early stages of colony development. The use of *pyr2* as a bidirectional marker has not previously been reported for *T. reesei*, but we here demonstrate that it serves as a highly reliable alternative to the previously used *pyr4* gene [8], encoding orotidine-5′-monophosphate decarboxylase. The applicability of the expression system was confirmed by expression of a *Thermomyces lanuginosus* lipase as reporter protein.

## Results and discussion

### Construction of a *tku70*∆ *pyr2*∆ strain

First, the *tku70* gene involved in NHEJ was disrupted in the original *T. reesei* QM6a isolate by transforming this strain with a gene-targeting substrate based on the *amd*S marker; see Materials and methods for details. Sixty transformants were obtained and a subsequent PCR analysis (data not shown) identified a single transformant containing the desired disruption of *tku70*. The correct integration of *amdS* into *tku70* in this transformant was confirmed by Southern blotting (Additional file 1: Figure S1) and the resulting strain was named MJ-T-001.

### *pyr2* as a bidirectional marker

**Figure 1:**
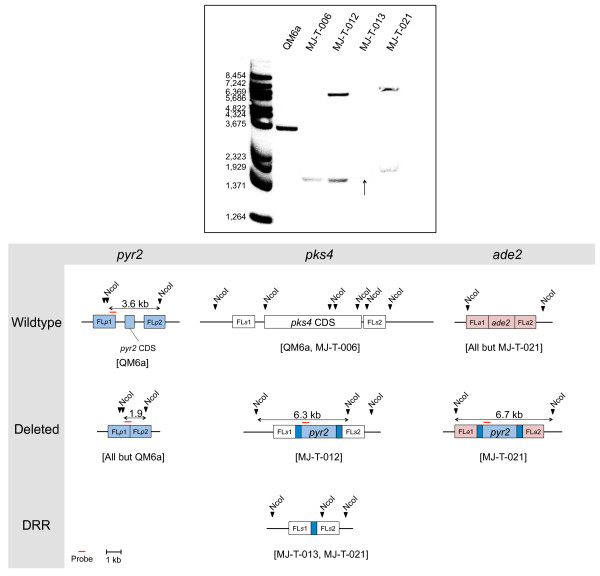
**Southern blot for confirmation of correct deletion of *pyr2*, *pks4 *and *ade2*.** NcoI was used for all digestions together with a 485 bp probe targeting the *pyr2* promoter and FL*p*1. The probe was amplified using primer pair *pyr2*prom-P-fw and *pyr2*prom-P-rv. An arrow indicates a very faint band for MJ-T-013. An enhanced version of the picture is provided as Additional file 2: Figure S2. The three affected loci and expected band sizes are seen underneath the Southern blot picture. Marker: BstII digested λ DNA. DRR: Direct repeat recombinant, FL*a*1: Upstream *ade2* flank, FL*a*2: Downstream *ade2* flank, FL*p*1: Upstream *pyr2* flank, FL*p*2: Downstream *pyr2* flank, FL*s*1: Upstream *pks4* flank, FL*s*2: Downstream *pks4* flank.

Orthologous genes of the *pyr2* gene have been used as bidirectional markers in several species such as *Candida guillermondii*, *Schwanniomyces alluvius* and *Haloferax volcanii*[9-11] based on the principle that 5-Fluoroorotic acid (5-FOA) is metabolized to 5-Fluorodeoxyuridine monophosphate (5-FdUMP), a suicide inhibitor of thymidylate synthase essential for DNA synthesis, in a process that depends on the orotate phosphoribosyl transferase activity of PYR2 [12,13]. To investigate the possibility that *pyr2* could also be used as a bidirectional marker in *T. reesei*, we deleted the entire coding sequence (CDS) of *pyr2* (DDBJ/EMBL/GenBank protein accession number: EGR51642.1, JGI ID: 21435) in MJ-T-001. In this case, the gene-targeting substrate did not contain a selectable marker as we hypothesized that correctly targeted strains, unlike wild-type strains, would survive exposure to 5-FOA. Eleven transformants were obtained after transformation with the *pyr2* deletion construct on 5-FOA plates, but only three of these displayed normal growth rates. As expected for correctly targeted strains these three transformants were all uridine auxotroph as they required addition of uridine to the media in order to grow [14-16]. Finally, deletion of *pyr2* was confirmed in one of the three transformants by PCR (data not shown) and by Southern blotting (Figure 1 and Additional file 2: Figure S2). The resulting strain was named MJ-T-006.

**Figure 2:**
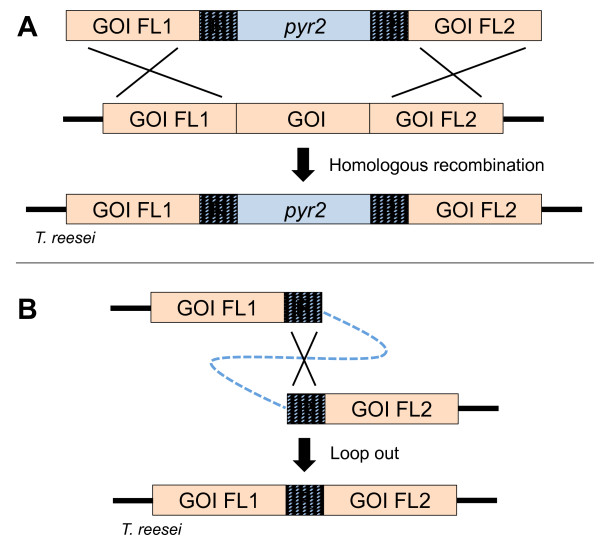
**Use of *pyr2 *for genetic deletion and direct repeat recombination. A**: Genetic deletion using *pyr2* as selectable marker. **B**: Elimination of *pyr2* by direct repeat recombination, selected for by subjection to 5-FOA. GOI: Gene of interest, FL1: Upstream flank, FL2: Downstream flank, R: Direct repeats.

Two gene-targeting substrates were constructed to determine whether *pyr2* could serve as bidirectional marker. One was designed to eliminate *pks4* (DDBJ/EMBL/GenBank protein accession number: EGR44538.1, JGI ID: 82208), a gene that encodes the polyketide synthase necessary for production of green conidial pigment [17]; and *ade2* (DDBJ/EMBL/GenBank protein accession number: EGR49709.1, JGI ID: 105832), an ortholog of the *ade2* gene in *Saccharomyces cerevisiae*, which encodes a phosphoribosylaminoimidazole carboxylase necessary for production of purines. Lack of this enzyme in species such as *S. cerevisiae, Pichia pastoris* and *Aspergillus oryzae*[18-20] results not only in adenine auxotrophy, but also in development of red colonies as the precursor 5-aminoimidazole ribonucleotide accumulates in the cell and polymerizes [21]. Both gene targeting substrates were based on a *pyr2* selectable marker, which was flanked by 375 bp direct repeats, originating from the *A. oryzae pyr4* promotor region, to allow for subsequent marker excision by direct repeat recombination, see Figure 2.

**Figure 3:**
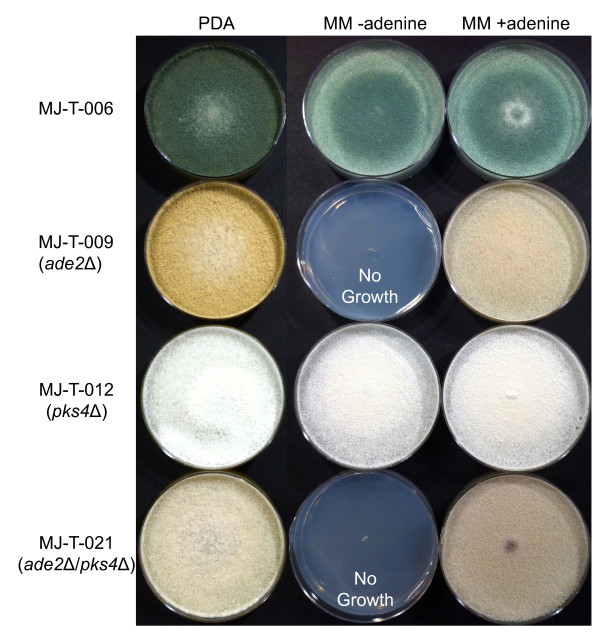
**Phenotypical differences between selected deletion strains obtained using *pyr2 *as selective marker.** Three media types were used, the rich media PDA, and MM with and without supplemented adenine. It is seen that MJ-T-006 is dark green (as the wild-type) while the other mutant strains all have variations in their colorations. MJ-T-009 (*ade2*Δ) has a yellow coloration and is at the same time adenine auxotroph, MJ-T-012 (*pks4*Δ) has a white coloration and can grow in absence of adenine while MJ-T-021 (*ade2*Δ/*pks4*Δ) has a light brown coloration and is also adenine auxotroph.

Transformation of the *pks4* gene-targeting substrate into MJ-T-006 produced 29 uracil prototroph transformants on Trichoderma minimal medium (MM) plates. After the transformants were transferred to PDA plates for sporulation, we observed that eleven (~40%) of the transformants lacked pigment production and were white as expected for *pks4* deleted strains (Figure 3). This strongly indicates that *pyr2* is an efficient marker for positive selection. In agreement with this view, PCR analysis of the white transformants confirmed that *pks4* was indeed deleted in all cases. Moreover, one of the white transformants, named MJ-T-012, was also confirmed by Southern blotting (Figure 1). The core of the gene targeting substrate contains *pyr2* flanked by direct repeats and the *pyr2* marker can therefore be eliminated by direct repeat recombination. To demonstrate that the *pyr2* gene can also be counter-selected, approximately 10^8^ spores from this strain were inoculated on plates supplemented with 5-FOA and uridine. Four colonies were isolated after five days of incubation and subsequent PCR- and sequencing analyses demonstrated that the *pyr2* gene was lost in all four strains as the result of recombination between the direct repeats. In agreement with these results, the four strains were also displaying a uridine auxotrophic phenotype. One of these colonies was named MJ-T-013 and used for further experiments.

To demonstrate that *pyr2* can be used as a marker for iterative gene targeting, we transformed MJ-T-013 with a gene targeting substrate designed to delete *ade2*. Nine uracil prototrophic transformants appeared on the primary transformation plates after transformation. Of these nine transformants, four developed orange/red coloration after four days of growth, indicating successful deletion of the *ade2* gene (Figure 3). All nine colonies were tested for adenine auxotrophy and as expected, the four red colonies were all adenine auxotrophs whereas the remaining five were not. Correct deletion of *ade2* in the four selected red transformants was confirmed by PCR (data not shown). One of the four transformants containing the *pks4* and *ade2* double deletion was verified by Southern blotting (Figure 1) and named MJ-T-021. Successful construction of *pks4* and *ade2* double deletion strains demonstrate that *pyr2* can be used as an alternative to *pyr4* for iterative gene targeting. Importantly, the coding sequence of *pyr2* is 435 bp shorter than that of *pyr4*, and as a consequence episomal vectors and gene targeting substrates that are based on *pyr2* will be smaller than if they are based on *pyr4*, which is often advantageous.

### A versatile integration vector for gene expression at *ade2*

**Figure 4:**
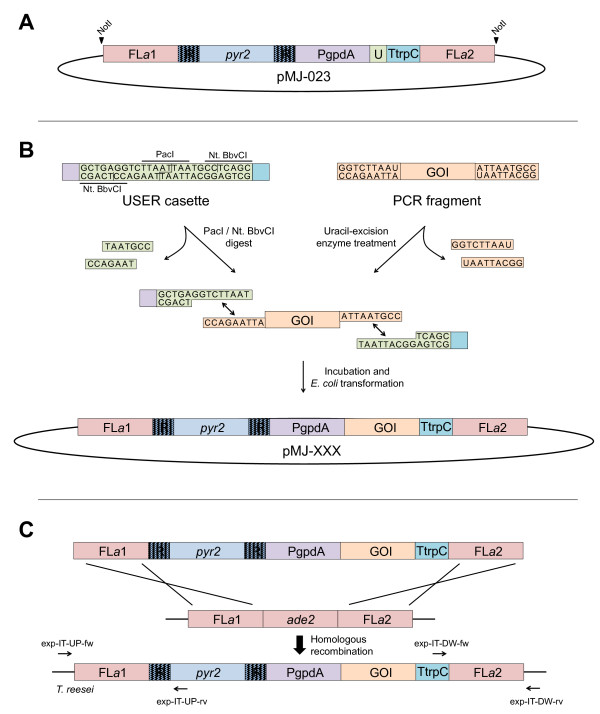
**A versatile integration vector to facilitate gene expression in the *ade2 *locus. A)** The pMJ-023 vector contains gene-targeting sequences (FL*a*1: upstream region of *ade2* and FL*a*2*:* downstream region of *ade2*) for integration into *ade2* locus (in pink); a *pyr2* selectable/counter selectable marker (in light blue) flanked by direct repeats (in striped blue); and a USER cassette (in green) flanked by the PgpdA promoter (in purple) and the TrpC terminator (in turquois). The NotI cut sites that are used for excising the gene-targeting substrate are indicated. The *E. coli* vector backbone is marked in black; see Materials and methods for more information. **B)** The gene of interest (GOI) (orange) is inserted into the vector by USER cloning. **C)** The liberated gene-targeting substrate containing the GOI expression cassette integrates into *ade2* by homologous recombination, in a process that results in *ade2* deletion. Primer targets for diagnostic PCRs are indicated.

In agreement with other studies, e.g. [6,7,14-16,22], we find that the gene targeting efficiency is quite high in strains with defective NHEJ. For both *pks4* and *ade2,* we obtained deletions with an efficiency of ~40%. Although this success rate is sufficient for small-scale experiments, it may hamper using *T. reesei* as a high-throughput screening platform for the discovery and production of novel enzymes. However, the fact that *ade2* deletion strains rapidly develop red color as they grow on MM plates may simplify screening. Specifically, we envisioned that by inserting genes for heterologous expression into *ade2*, correctly targeted transformants can be easily selected as red colonies. Importantly, we find that deletion of *ade2* does not have a negative impact on the growth rate on MM medium supplemented with adenine as judged by race tube experiments (Table 1); and the locus is therefore well-suited for gene insertion and expression. We therefore decided to exploit the red/white color-screen offered by the *ade2* locus and incorporate it as a central part of our expression platform. Consequently, we constructed a versatile integration vector, pMJ-023, see Figure 4A, which in a single cloning step equips a gene of interest (GOI) with all necessary parts for integration and expression from *ade2*. Hence, the vector contains gene-targeting sequences for integration into *ade2* and the *pyr2* marker for selection/counter selection. Moreover, it contains a USER cloning cassette [23,24] flanked by the *Aspergillus nidulans* PgpdA promoter and a TtrpC terminator to allow for gene expression. Insertion of a GOI into the USER cassette by USER cloning or USER fusion, see Figure 4B, not only facilitates highly efficient vector construction in a manner compatible with high-throughput experiments, but also offers a simple routine for introducing additional genetic manipulations like site-directed mutagenesis, or addition of tagging sequences encoding epitope-, GFP-, or purification tags. A simple restriction enzyme digest of the resulting vector liberates the gene-targeting substrate for integration into *ade2* by homologous recombination, see Figure 4C.

**Table 1:** Growth rates of mutant strains in MM supplemented with 0.5 mM adenine

Strain	MJ-T-001	MJ-T-009	MJ-T-021
Genotype	*pyr2*	*pyr2*Δ *ade2*Δ::*pyr2*	*pyr2*Δ *pks4*Δ *ade2*Δ::*pyr2*
Growth rate (mm/h)	0.91 (st.dev. 0.04)	0.99 (st.dev. 0.02)	0.91 (st.dev. 0.09)

All strains are *tku70::amdS*. Genetic differences in genotype are as indicated.

To demonstrate this concept we decided to express the *T. lanuginosus* lipase gene, *lip*, from the *ade2* locus. Accordingly, *lip* was positioned between the *A. nidulans* PgpdA promoter and TtrpC terminator in pMJ-023, resulting in pMJ-051. The gene-targeting substrate containing the PgpdA::*lip*::TtrpC fragment was liberated from the vector backbone by restriction enzyme digestion and inserted into *ade2* using *pyr2* as selection marker. After transformation, several red colonies appeared and correct insertion of the expression construct in three of these transformants was confirmed by PCR and Southern blot analyses (Additional file 3: Figure S3). The three transformants, MJ-T-033-1, -2 and −3, were analyzed for *T. lanuginosus* lipase production using a well-developed assay for measuring lipase activity, see Materials and methods. In a parallel experiment, an empty expression construct containing PgpdA::TtrpC was inserted into *ade2* to generate strain MJ-T-020, which was analyzed in the same manner. The average lipase activities (Lipase Units, LU) for MJ-T-020, MJ-T-033-1, -2 and -3 were 1.19 LU ± 0.05, 2.00 LU ± 0.15, 1.85 LU ± 0.09 and 2.09 LU ± 0.15 pr. 10 μl supernatant, respectively. Hence, a significant and reproducible increase in the amounts of lipase activity could be detected in the strains expressing the PgpdA controlled *lip*. Specifically, additional lipase activity corresponding to an average of 0.79 LU was produced in these strains.

## Conclusions

In summary, we have developed an expression platform, which is designed for high-throughput construction of defined integrated *T. reesei* strains suitable for setup of large expression studies. The compatibility of USER cloning with PCR enables easy site-directed mutagenesis, promoter swaps, and epitope- and GFP tagging for protein engineering, characterization, purification, and production. Moreover, selected mutants can be further analyzed/optimized by iterative gene targeting using *pyr2* as a bidirectional selective marker.

## Materials and methods

### Strains and media

The strains included in this study are listed in Table 2. Yeast extract-peptone-dextrose and Trichoderma Minimal Media (MM) was made as in Fink & Hicks [25] and Gruber et al., 1990 [26], respectively. For composition of MM including additional supplements, see Additional file 4: Table S1. Potato dextrose agar (PDA) was obtained from BD/Difco.

**Table 2:** Overview of the strains used in this study

**Strain**	**Genotype**	**Growth in presence of 5-FOA**	**Prototrophy**		**Reference**
			**Uridine**	**Adenine**	
QM6a	WT	-	+	+	(Martinez et al., 2008) [3]
MJ-T-001	*tku70*^-^::*amd*S^+^	-	+	+	This study
MJ-T-006	*tku70*^-^::*amd*S^+^*pyr2*Δ	+	-	+	This study
MJ-T-009	*tku70*^-^::*amd*S^+^*pyr2*Δ *ade2*Δ::*pyr2*	-	+	-	This study
MJ-T-012	*tku70*^-^::*amd*S^+^*pyr2*Δ *pks4*Δ::*pyr2*	-	+	+	This study
MJ-T-013	*tku70*^-^::*amd*S^+^*pyr2*Δ *pks4*Δ	+	-	+	This study
MJ-T-020	*tku70*^-^::*amd*S^+^*pyr2*Δ *ade2*Δ::PgpdA-TtrpC*-pyr2*	-	+	-	This study
MJ-T-021	*tku70*^-^::*amd*S^+^*pyr2*Δ *pks4*Δ::*pyr2*Δ *ade2*Δ::*pyr2*	-	+	-	This study
MJ-T-033	*tku70*^-^::*amd*S^+^*pyr2*Δ *ade2*Δ::PgpdA-*lip-*TtrpC-*pyr2*	-	+	-	This study

5-FOA: 5-Fluoorotic acid.

The genotypes are listed together with selected phenotypic characteristics.

### Gene identification

The putative *T. reesei* proteins encoded by *ade2* (JGI ID: 105832), *pyr2* (JGI ID: 21435) and *pks4* (JGI ID: 82208) were identified by BLASTing i) the 250 amino acid residues (aa) of the *A. oryzae* PyrF protein (/EMBL/GenBank protein accession number XM_001821908.2), ii) the 2157 aa sequence of the *A. nidulans* Ywa1 protein and iii) the 571 aa sequence of the *S. cerevisiae* Ade2 protein*,* respectively, in the JGI *T. reesei* filtered model proteins database (http://genome.jgi-psf.org/pages/blast.jsf?db=Trire2). The corresponding gene sequences were subsequently extracted from the same source.

### Vector construction

An overview of the primers used for vector construction and the resulting plasmids are listed in Additional file 5: Table S2 and Additional file 6: Table S3, respectively. PCR fragments used for the constructions were made by using PfuX7 polymerase [27] and purified from agarose gel using the GFX kit (GE healthcare, Little Chalfont, United Kingdom). Specifically, pMJ-001 was made by inserting the relevant PCR fragment (see Additional file 7: Figure S4 and Additional file 5: Table S2) into pCR2.1 (Invitrogen) by topoisomerase-mediated ligation according to the instructions provided by the manufacturer. pMJ-005 was made by inserting the relevant PCR fragment into a StuI and SnaBI vector fragment of pMJ-001 using In-Fusion® ligation (Clontech). The remaining vectors were made by fusing PCR fragments with the pU1111-1 vector backbone [23] by restriction enzyme and ligase independent uracil-excision cloning [24] using the USER™ Friendly Cloning Kit protocol (New England Biolabs), see Additional file 5: Table S2 and Additional file 6: Table S3 for details. All vectors were cloned by transformation into chemically competent Fusion-Blue *E. coli* cells (Clontech, Mountain View, USA) as described in the In-Fusion® Dry-Down PCR Cloning Kit Protocol-at-a-Glance.

### Strain construction

*T. reesei* protoplastations and transformations were performed as described by Gruber and co-workers [26]. Approximately 10 μg linearized DNA by restriction endonucleases was used for each transformation. Correct targeted integration was verified by diagnostic PCRs and Southern blots; see primers in Additional file 8: Table S4. All strains were incubated on solid media for five days at 28°C; and in liquid media for 48 hours at 30°C and 200 RPM. The exception was for lipase assays as described later.

The gene targeting substrate for construction of MJ-T-001 was obtained by digesting pMJ-005 with ClaI. This fragment was transformed into QM6a and transformants selected by using the *amdS* selection marker on MM plates supplemented with acetamide. All remaining gene-targeting substrates were liberated from the plasmid backbone by NotI digestion and gel purified. MJ-T-006 was generated by transforming MJ-T-001 with the gene targeting substrate obtained from pMJ-017. In this case transformants were selected by plating on MM plates containing 5-FOA and uridine. MJ-T-009 and MJ-T-012 was obtained by transforming MJ-T-006 with gene targeting substrates isolated from pMJ-031 and pMJ-030, respectively. MJ-T-009 was selected on MM supplemented with adenine and MJ-T-012 on MM. MJ-T-013 was generated from MJ-T-012 by selecting for recombinants where the *pyr*2 marker has been lost by direct repeat recombination. Specifically recombinants were selected by plating 10^8^ spores on 5-FOA MM plates.

The *pyr2* loop out strain, MJ-T-013, was transformed with the gene targeting substrate isolated from pMJ-031 and transformants selected on MM plates to acquire MJ-T-021. MJ-T-020 was obtained by transforming MJ-T-006 with the gene targeting substrate isolated from pMJ-023 and plating on MM plates. After transforming MJ-T-006 with the gene targeting substrate originating from pMJ-051, MJ-T-033-1, -2 and −3 were isolated from MM plates.

All transformants were streak-purified on the proper selection medium before further analysis. All strains were verified by diagnostic PCR and Southern blot, Figure 1, Additional file 1: Figure S1, Additional file 3: Figure S3 and Additional file 8: Table S4.

### Growth rate measurements

The linear growth rates of MJ-T-001, MJ-T-009, MJ-T-012 and MJ-T-021 were measured in race tubes, as described by White and Woodward [28].

### Expression of *T. lanuginosus* lipase from the *ade2* site

The *T. lanuginosus* lipase gene (*lip*) (accession no. AF054513) was kindly provided by Jan Lehmbeck, Novozymes, Bagsvaerd. The lipase was produced by growing MJ-T-033-1, -2 and -3 in 10 ml YPD at 30°C for four days at 200 rpm. At this point, the amount of lipase in the medium was determined by measuring the esterase activity. Specifically, the rate of p-nitrophenol formation using *p*-nitrophenyl valerat as substrate was measured in microtiter plate wells by mixing 10 μl supernatant, 20 μl dilution buffer (50 mM Tris/HCL (pH 7.5), 10 mM CaCl_2_ and 0.075% Brij-35 (Thermo Fisher Scientific, Rockford, USA)) and 200 μl substrate solution (0.6 mM 4-Nitrophenyl valerate (Sigma N4377) dissolved in methanol).

The Lipase Units (LU) were measured in an ELISA reader as absorption at 405 nm (peak absorbance of *p*-nitrophenol) in 30-second intervals for 40 minutes. The amount of LU in the samples was calculated based on included standards.

## Competing interests

The authors declare that they have no competing interests.

## Authors’ contributions

MSJ has conducted all the experiments regarding this manuscript and has analyzed all data. The initial manuscript draft was composed by MSJ. DAS, PFJ and UHM have provided input for setup of the experimental design and have helped finalizing the manuscript. All authors read and approved the final manuscript.

## Supplementary Material

Additional file 1: Figure S1 Southern blot for confirmation of *tku70* truncation. XbaI was used for all digestions. The 900 bp probe targeting the *tku70* CDS was amplified using primers *tku70*-P-fw and *tku70*-P-rv. Marker: BstII digested lambda DNA. Click here for file

Additional file 2: Figure S2 Enhanced version of Figure 1. An arrow indicates the band for MJ-T-013 that was not visible in Figure 1. Click here for file

Additional file 3: Figure S3 Southern blots for confirmation of correct insertion of the *lip* expression construct. BamHI and NheI were used for all digestions. A: Southern blot using two probes. One probe targeting FL*a*1 and the other FL*a*2. The 1601 bp FL*a*1 probe was amplified with primers *ade2*-P-UP-fw and *ade2*-P-UP-rv and the 1567 bp FL*a*2 probe was amplified using primers *ade2*-P-DW-fw and *ade2*-P-DW-rv B: Southern blot using a probe targeting *lip*. The 876 bp probe was amplified using the primers lipase-P-fw and lipase-P-rv. Marker: BstII digested lambda DNA. FL*a*1: Upstream *ade2* flank, FL*a*2: Downstream *ade2* flank, *lip*: *T. lanuginosus* lipase gene, T: TtrpC terminator. Click here for file

Additional file 4: Table S1 Additional media supplements. The supplements added to MM plates according to specific genotypes. Click here for file

Additional file 5: Table S2 Oligonucleotides used for production of PCR fragments used for vector construction. Click here for file

Additional file 6: Table S3 Plasmids constructed in this study. Click here for file

Additional file 7: Figure S4 Construction of pMJ-005. 1. A 2311 bp *T. reesei tku70* gene fragment was amplified with the primers *tku70*-fw and *tku70*-rv. 2. The fragment was introduced into TOPO® vector pCR2.1 (invitrogen) by following the manufacturers TOPO® cloning protocol, resulting in pMJ-001. 3. The pMJ-001 construct was linearized by digestion with StuI and SnaBI, digesting after the 1154th and 1171th bp of the *tku70* coding sequence (CDS), respectively. 4. A 2762 bp amplicon of the *Aspergillus nidulans* acetamidase gene (*amd*S) was obtained by PCR with primers *amd*S-fw and *amd*S-rv and used as selective marker. The two primers each carried a 15 bp 5′-end sequence complimentary to one end of the linear pMJ-001 vector. 5. The PCR product was cloned into pMJ-001 by In-Fusion® cloning (Clontech) by following the manufacturer’s protocol, resulting in pMJ-005. The construct contains the *amd*S selective marker flanked by *tku70* fragments of 1.3 and 1.0 kb in the pCR2.1 topo vector backbone. Click here for file

Additional file 8: Table S4 Oligonucleotides used for diagnostic PCR, and Southern blot probes. Click here for file
