# Supplementary figures and images for: A novel platform for heterologous gene expression in Trichoderma reesei (Teleomorph Hypocrea jecorina)
Source: Microb Cell Fact. 2014 Mar 6;13:33. doi: 10.1186/1475-2859-13-33 (PMC4015775; doi:10.1186/1475-2859-13-33)

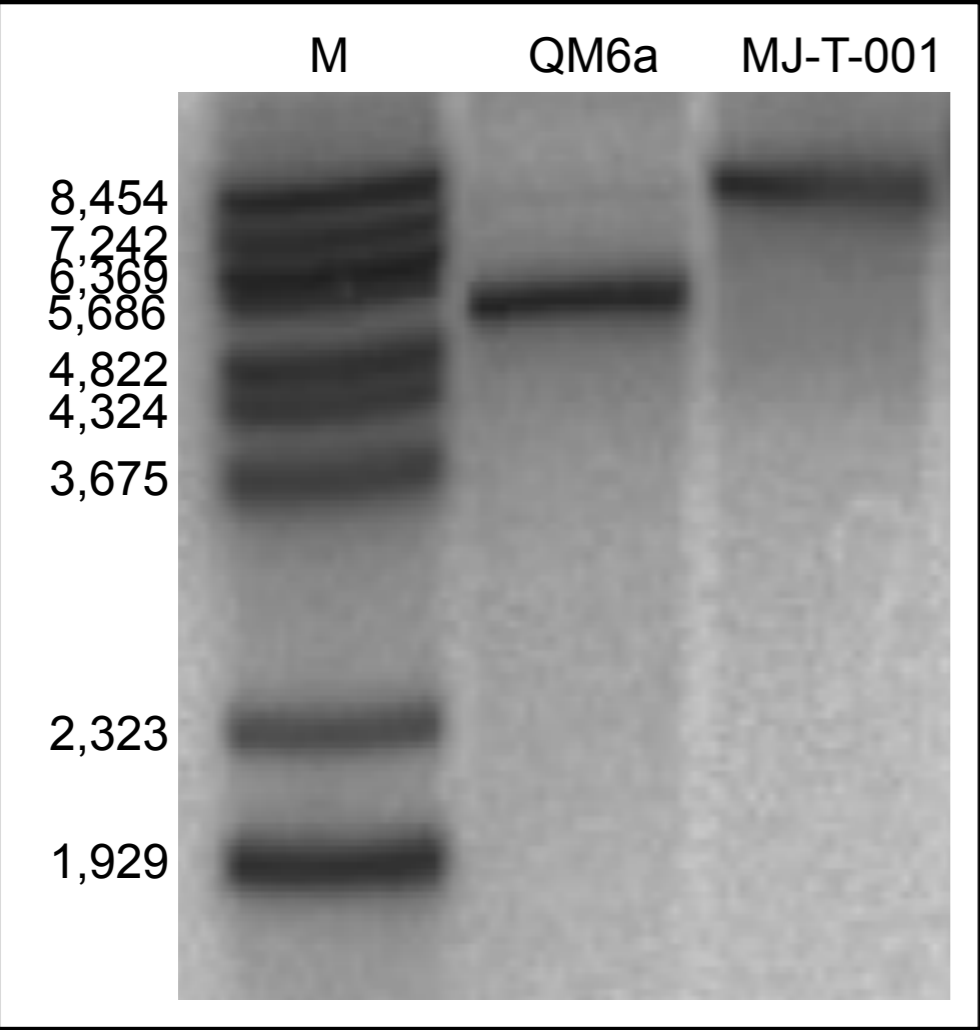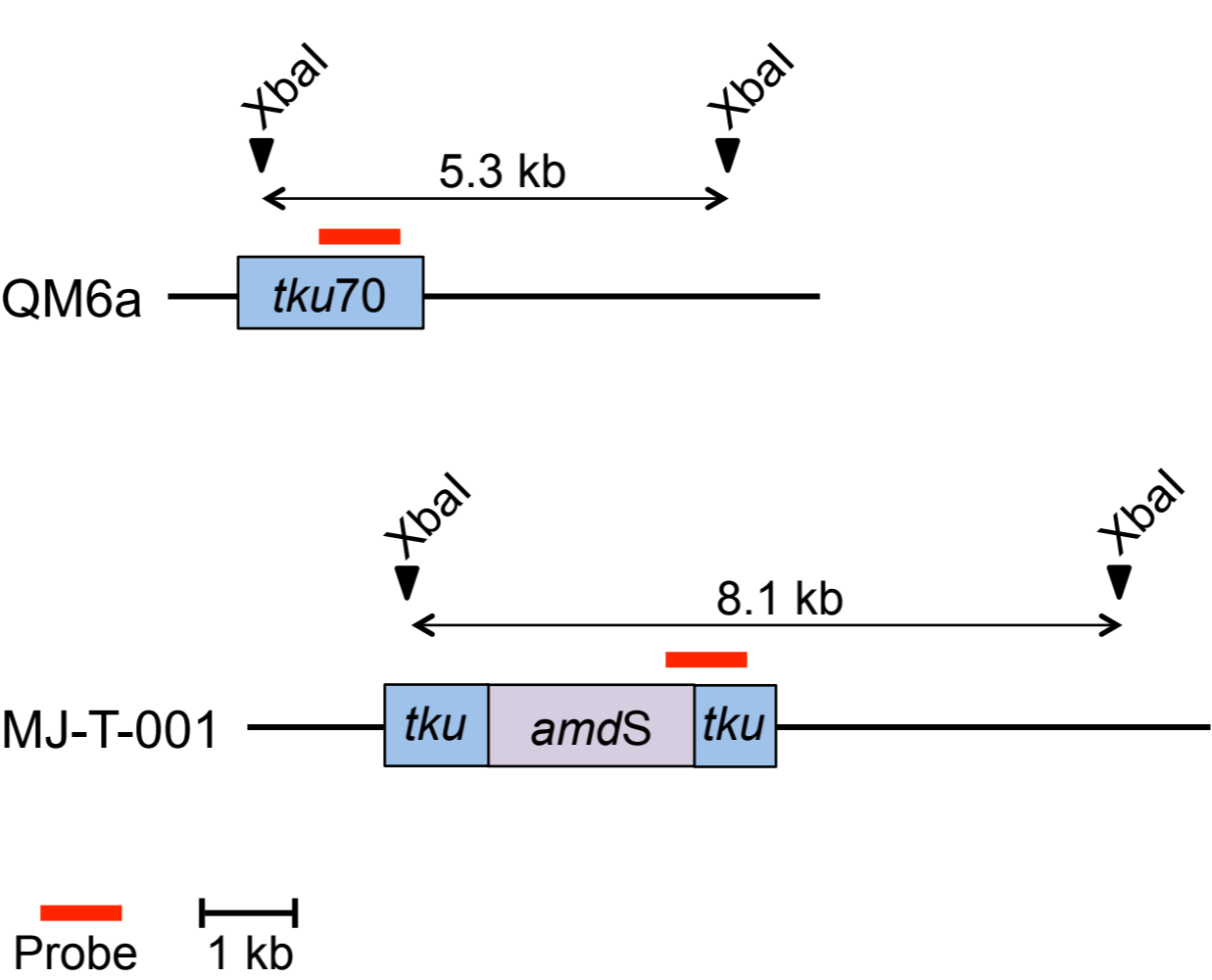

Supplement: Additional file 1: Figure S1 — Southern blot for confirmation of tku70 truncation. XbaI was used for all digestions. The 900 bp probe targeting the tku70 CDS was amplified using primers tku70-P-fw and tku70-P-rv. Marker: BstII digested lambda DNA. [file 1475-2859-13-33-S1.pdf]

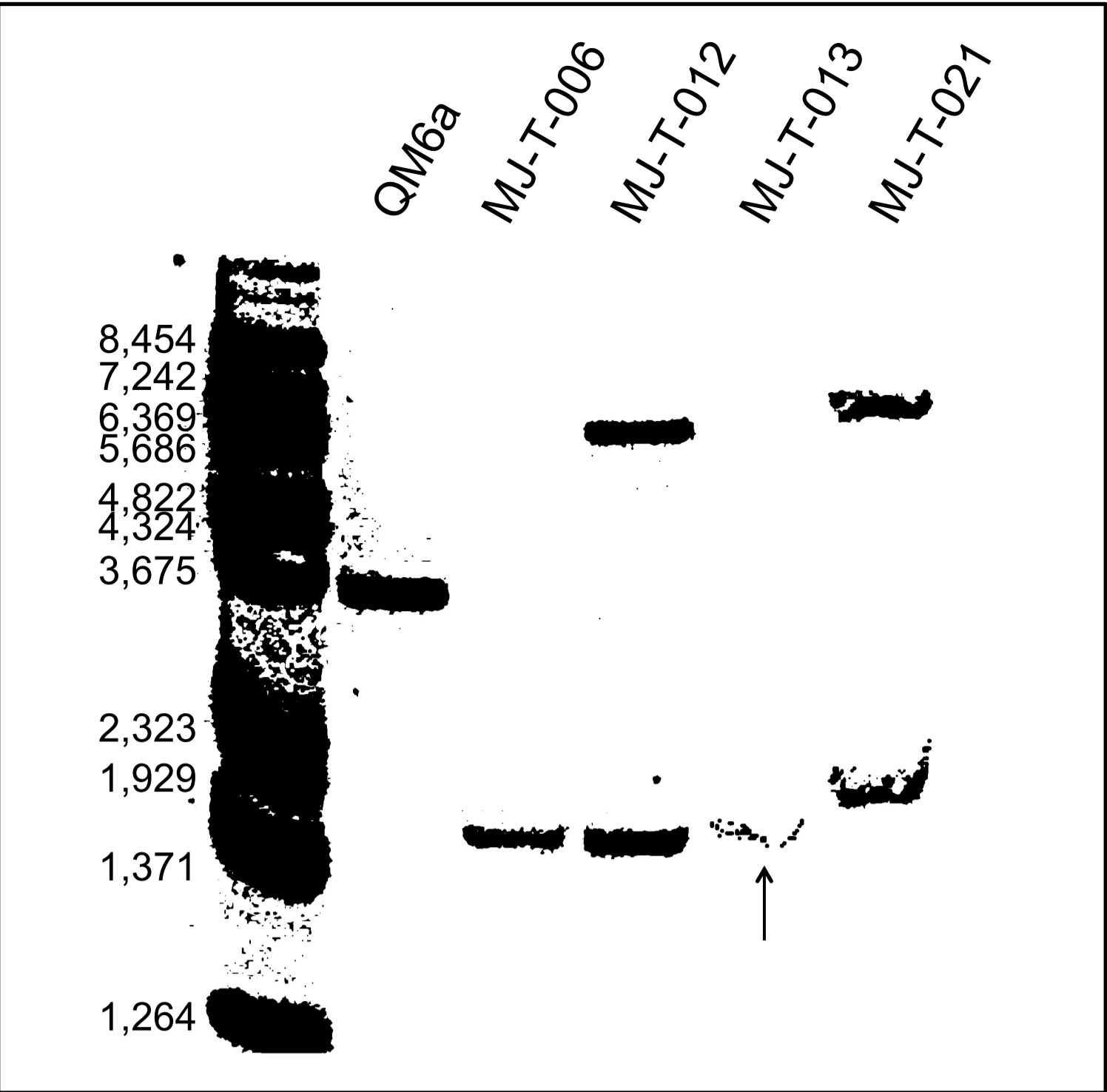

Supplement: Additional file 2: Figure S2 — Enhanced version of Figure 1. An arrow indicates the band for MJ-T-013 that was not visible in Figure 1. [file 1475-2859-13-33-S2.pdf]

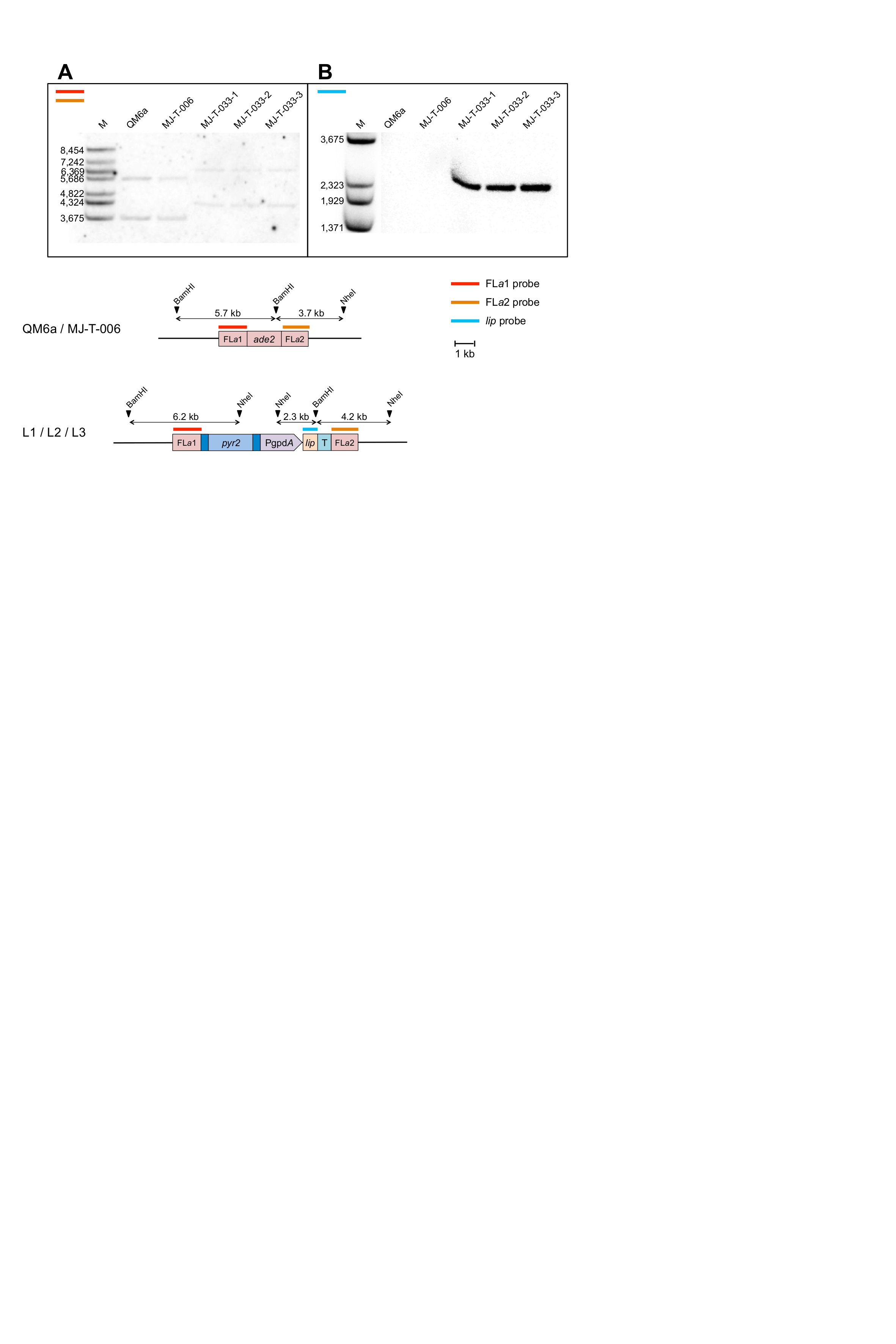

Supplement: Additional file 3: Figure S3 — Southern blots for confirmation of correct insertion of the lip expression construct. BamHI and NheI were used for all digestions. A: Southern blot using two probes. One probe targeting FLa1 and the other FLa2. The 1601 bp FLa1 probe was amplified with primers ade2-P-UP-fw and ade2-P-UP-rv and the 1567 bp FLa2 probe was amplified using primers ade2-P-DW-fw and ade2-P-DW-rv B: Southern blot using a probe targeting lip. The 876 bp probe was amplified using the primers lipase-P-fw and lipase-P-rv. Marker: BstII digested lambda DNA. FLa1: Upstream ade2 flank, FLa2: Downstream ade2 flank, lip: T. lanuginosus lipase gene, T: TtrpC terminator. [file 1475-2859-13-33-S3.jpeg]

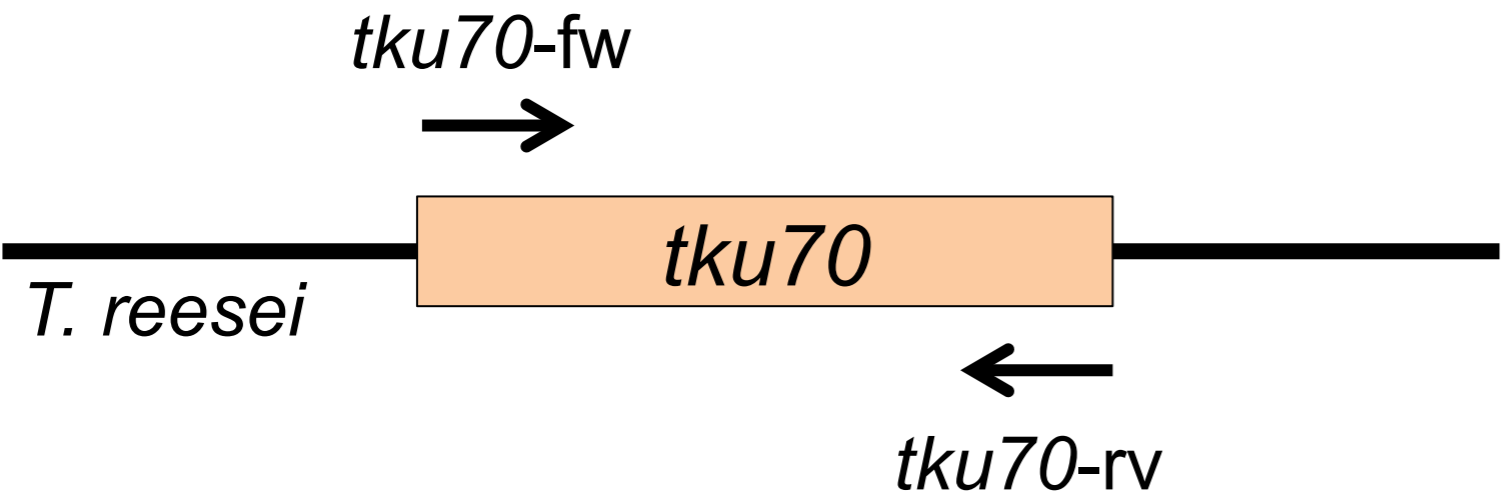

1 ↓

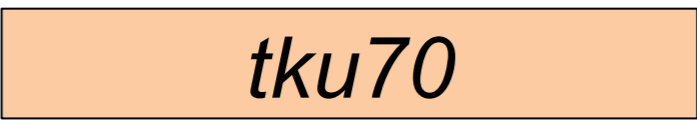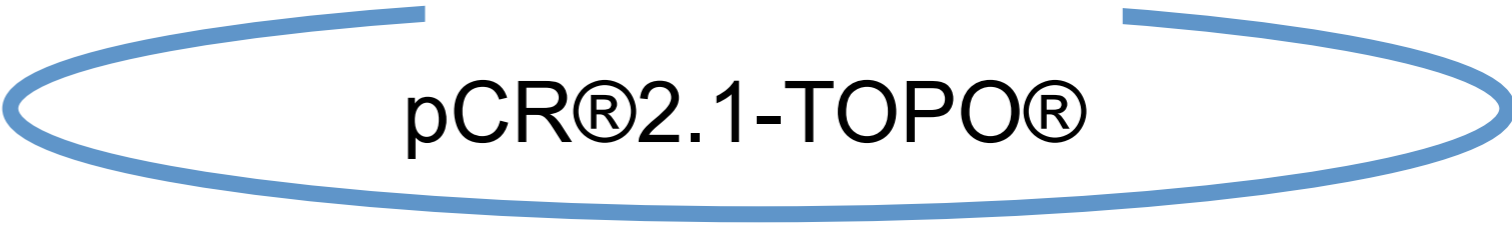

2 ↘

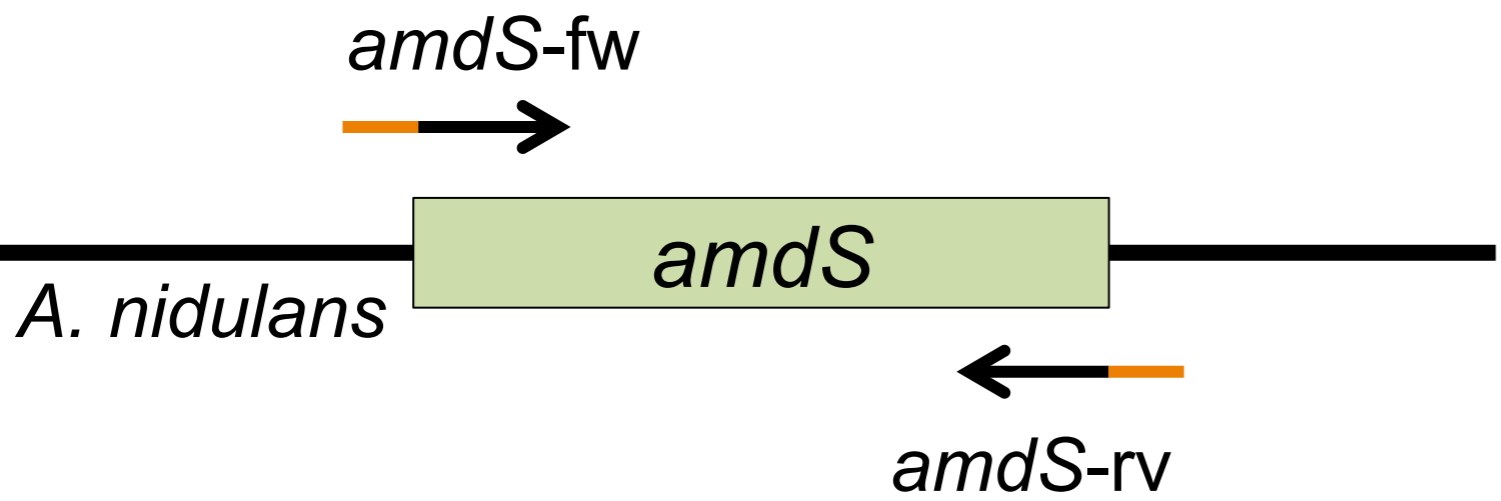

4 ↓

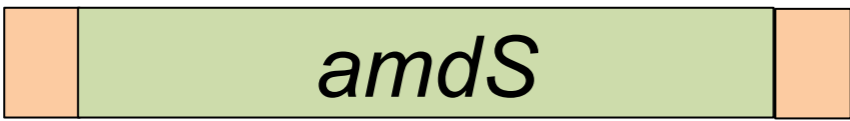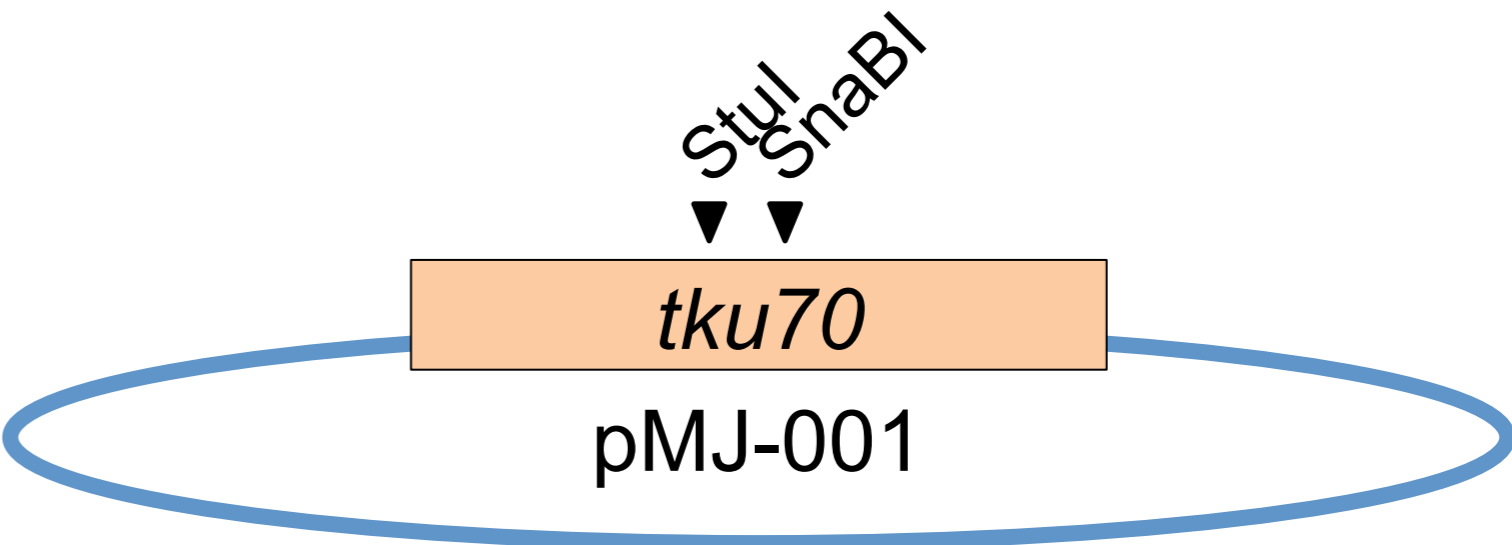

3 ↓

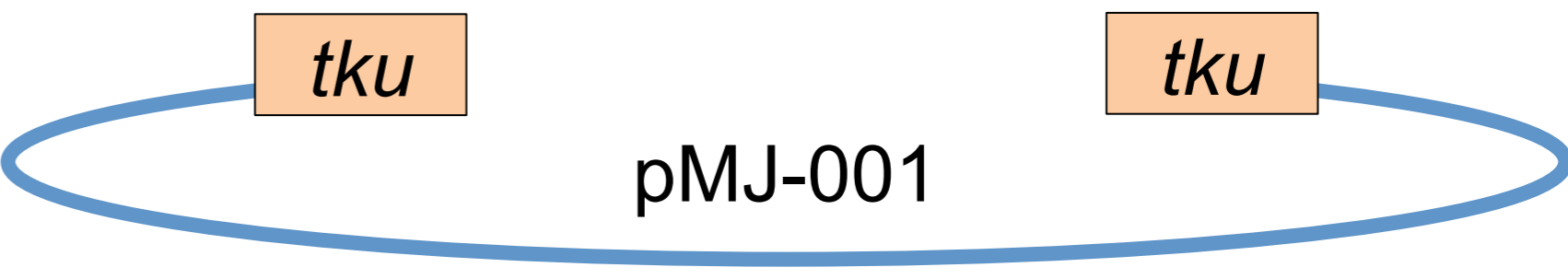

5 ↘

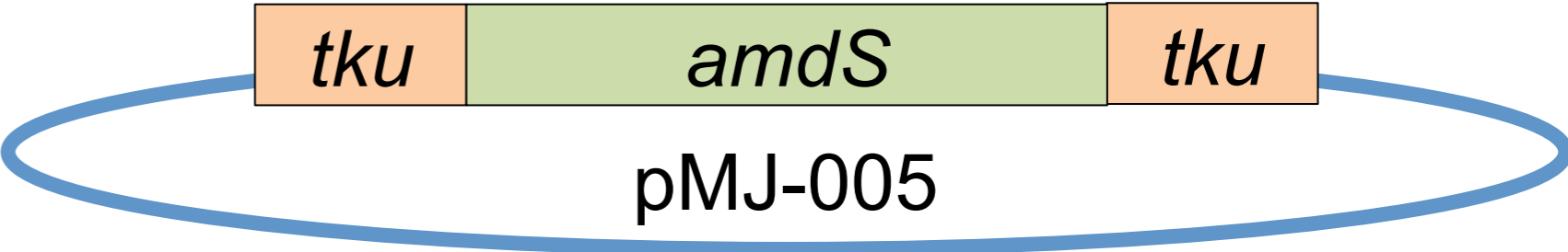

Supplement: Additional file 7: Figure S4 — Construction of pMJ-005. 1. A 2311 bp T. reesei tku70 gene fragment was amplified with the primers tku70-fw and tku70-rv. 2. The fragment was introduced into TOPO® vector pCR2.1 (invitrogen) by following the manufacturers TOPO® cloning protocol, resulting in pMJ-001. 3. The pMJ-001 construct was linearized by digestion with StuI and SnaBI, digesting after the 1154th and 1171th bp of the tku70 coding sequence (CDS), respectively. 4. A 2762 bp amplicon of the Aspergillus nidulans acetamidase gene (amdS) was obtained by PCR with primers amdS-fw and amdS-rv and used as selective marker. The two primers each carried a 15 bp 5′-end sequence complimentary to one end of the linear pMJ-001 vector. 5. The PCR product was cloned into pMJ-001 by In-Fusion® cloning (Clontech) by following the manufacturer’s protocol, resulting in pMJ-005. The construct contains the amdS selective marker flanked by tku70 fragments of 1.3 and 1.0 kb in the pCR2.1 topo vector backbone. [file 1475-2859-13-33-S7.pdf]
