# Supplementary material for: A novel platform for heterologous gene expression in Trichoderma reesei (Teleomorph Hypocrea jecorina)
Source: Microb Cell Fact. 2014 Mar 6;13:33. doi: 10.1186/1475-2859-13-33 (PMC4015775; doi:10.1186/1475-2859-13-33)
Supplement: Additional file 4: Table S1 — Additional media supplements. The supplements added to MM plates according to specific genotypes. [file 1475-2859-13-33-S4.doc]

| **Table S1. Additional media supplements**. The supplements added to MM plates according to specific genotypes | | | | | |
| --- | --- | --- | --- | --- | --- |
|  |  | |  | Nitrogen source | |
| Genotype | 5-FOA  (1.5 mg/ml) | Uridine  (10mM) | Adenine  (0.5mM) | Acetamide  (10mM) | (NH_4_)_2_SO_4_  (50 mM) |
| *amd*S^+^ | - | - | - | + | - |
| *pyr2*^+^ | - | - | - | - | • |
| *pyr2*^-^ | + | • | - | - | • |
| *ade2*^-^ | - | - | • | - | • |
| -: not added, +: added for selection, •: needed supplement | | | | | |
