# Supplementary material for: A novel platform for heterologous gene expression in Trichoderma reesei (Teleomorph Hypocrea jecorina)
Source: Microb Cell Fact. 2014 Mar 6;13:33. doi: 10.1186/1475-2859-13-33 (PMC4015775; doi:10.1186/1475-2859-13-33)
Supplement: Additional file 5: Table S2 — Oligonucleotides used for production of PCR fragments used for vector construction. [file 1475-2859-13-33-S5.doc]

| **Table S2. Oligonucleotides used for production of PCR fragments used for vector construction** | | |  |  |
| --- | --- | --- | --- | --- |
| Primer name | Sequence (5’-3’) | Fragment | Used for construction of | Template |
| *tku70*-fw | CTCTCATCTTGCAAGTGAAT | *tku70* | pMJ-001 | *Trichoderma reesei* |
| *tku70*-rv | CGTCCATTTCGATTCCGCAT |
| *amd*S-fw | AAGAGATAAAAAAGGCCTTTCTACGCCAGGACCGAGCA | *amdS* | pMJ-005 | *Aspergillus nidulans* |
| *amd*S-rv | GGGCGTAAAGTATACGTAATGCATCTGGAAACGCAACC |
| Vector-fw | AGGCGTGCAUCCGCATCATC | pU1111-1 vector fragment | pMJ-017, pMJ-021,  pMJ-030, pMJ-031 | pU1111-1 |
| Vector-rv | ATCTGGGUCGTGGTCGATTGTG |
| *pyr2*-fw | AGGGCGUGGAGCTGGATGGATGGGC | *pyr2* | pMJ-021 | *Trichoderma reesei* |
| *pyr2*-rv | ACAGCCAUGCCGACGCTGCCAAGAAG |
| *pyr2*-FLUP-fw | ACCCAGAUAGGGCTGGACGTCCACATCG | FL*p*1 | pMJ-017 | *Trichoderma reesei* |
| *pyr2*-FLUP-rv | ATAGTCUCAGGCTTGTGCCAGCCATG |
| *pyr2*-FLDW-fw | AGACTAUGCCCCGGGCTGC | FL*p*2 | pMJ-017 | *Trichoderma reesei* |
| *pyr2*-FLDW-rv | ATGCACGCCUGTCCATGTGCCCTATCTGCCTG |
| Repeat-UP-fw | ACCCAGAUGTACCCTAAGGATAGGCCCTAATC | Direct repeat UP | pMJ-021 | *Aspergillus oryzae* |
| Repeat-UP-rv | ACGCCCUCTAGCGCGTGCGCTGTAG |
| Repeat-DW-fw | ATGGCTGUGTACCCTAAGGATAGGCCCTAATC | Direct repeat DW | pMJ-021 | *Aspergillus oryzae* |
| Repeat-DW-rv | ATGCACGCCUCTAGCGCGTGCGCTGTAG |
| *ade2*-FLUP-fw | AATTAAUGCCTCAGCGCTCAAGTGAGCGACGGCTC | FL*a*1 | pMJ-023 | *Trichoderma reesei* |
| *ade2*-FLUP-rv | ACCCAGAUGCGGCCGCCAACCCATGGCGTAGGGAGG |
| *pyr2*rep-UP-fw | ATTAATUAAGACCTCAGCCGCCAGCAGTGTCACAATCGAC | Pyr2 + direct repeats | pMJ-023 | pMJ-021 |
| *pyr2*rep-DW-rv | ATGCACGCCUACGCAGTGGTCACGGTCCG |
| PgpdA-fw | ACTGCGUCGAATGCGTGCGATAATTCCC | PgpdA/TtrpC cassette | pMJ-023 | pU1111-1 |
| TtrpC-rv | ACGCGAUGGGCGCTTACACAG |
| *ade2*-FLDW-fw | ATCGCGUGATCTGACGCTGCGAGCCAG | FL*a*2 | pMJ-023 | *Trichoderma reesei* |
| *ade2*-FLDW-rv | ATGCACGCCUCGGTGGTTTGAGCGTCTGCG |
| *pyr2*rep-fw | AAGACCUCAGCCGCCAGCAGTGTCACAATCGAC | *pyr*2 + direct repeats | pMJ-030, pMJ-031 | pMJ-021 |
| *pyr2*rep-rv | AGCGACGGCUCGGTGATTTC |
| *pks4*-FLUP-fw | ACCCAGAUGTCATTCGAGGCGACGCAAG | Fl*s*1 | pMJ-030 | *Trichoderma reesei* |
| *pks4*-FLUP-rv | AGGTCTUGCCCTGGACTGAAAACGGGTG |
| *pks4*-FLDW-fw | ACCACTGCGTUAGGCCGCCAATGCTCTTGAC | FL*s*2 | pMJ-030 | *Trichoderma reesei* |
| *pks4*-FLDW-rv | ATGCACGCCUCCGCTGGGTTGACTCGATTG |
| *ade2*-FL1-fw | ACCCAGAUCAACCCATGGCGTAGGGAGG | FL*a*1 | pMJ-031 | *Trichoderma reesei* |
| *ade2*-FL1-rv | AGGTCTUGCTCAAGTGAGCGACGGCTC |
| *ade2*-FL2-fw | ACCACTGCGTUGATCTGACGCTGCGAGCCAG | FL*a*2 | pMJ-031 | *Trichoderma reesei* |
| *ade2*-FL2-rv | ATGCACGCCUCGGTGGTTTGAGCGTCTGCG |
| Lipase-fw | AGAGCGAUATGAGGAGCTCCCTTGTGCTG | *lip* | pMJ-051 | *Thermomyces lanuginosus* |
| Lipase-rv | TCTGCGAUCTAAAGACATGTCCCAATTAACCCG |
| Red – 5’-end tails for In-Fusion® cloning, green – nucleotides for reconstruction of restriction sites, blue – uracil-containing 5’-end tails for uracil-excision cloning. Abbreviations: FL: Flank, UP: Upstream, DW: Downstream. FL*a*1: Upstream *ade2* flank, FL*a*2: Downstream *ade2* flank, FL*p*1: Upstream *pyr2* flank, FL*p*2: Downstream *pyr2* flank, Fl*s*1: Upstream *pks4* flank, FL*s*2: Downstream *pks4* flank. | | | | |
