# Supplementary material for: A novel platform for heterologous gene expression in Trichoderma reesei (Teleomorph Hypocrea jecorina)
Source: Microb Cell Fact. 2014 Mar 6;13:33. doi: 10.1186/1475-2859-13-33 (PMC4015775; doi:10.1186/1475-2859-13-33)
Supplement: Additional file 6: Table S3 — Plasmids constructed in this study. [file 1475-2859-13-33-S6.docx]

| **Table S3. Plasmids constructed in this study** | | | | |
| --- | --- | --- | --- | --- |
| Plasmid ID | Content | Vector | Fragments | Cloning method |
| pMJ-001 | *tku70* gene | pCR2.1 | *tku70* | TOPO® |
| pMJ-005 | Gene targeting substrate  *tku70* disruption | pMJ-001 | *amd*S | In-fusion® |
| pMJ-017 | Gene targeting substrate  *pyr2* deletion | pU1111-1 vector fragment | FL*p*1  FL*p*2 | uracil-excision |
| pMJ-021 | *pyr2* flanked by direct repeats | pU1111-1 vector fragment | Direct repeat UP  *pyr2* (gene/term/prom)  Direct repeat DW | uracil-excision |
| pMJ-023 | *ade2* targeting cassette for gene expression | pU1111-1 vector fragment | FL*a*1  *pyr2* + direct repeats  PgpdA/TtrpC cassette  FL*a*2 | uracil-excision |
| pMJ-030 | Gene targeting substrate  *pks4* deletion | pU1111-1 vector fragment | FL*s*1  *pyr*2 + direct repeats  FL*s*2 | uracil-excision |
| pMJ-031 | Gene targeting substrate  *ade2* deletion | pU1111-1 vector fragment | FL*a*1  *pyr*2 + direct repeats  FL*a*2 | uracil-excision |
| pMJ-051 | *lip* expression cassette | pMJ-023 | *lip* | uracil-excision |
| FL*a*1: Upstream *ade2* flank, FL*a*2: Downstream *ade2* flank, FL*p*1: Upstream *pyr2* flank, FL*p*2: Downstream *pyr2* flank, FL*s*1: Upstream *pks4* flank, FL*s*2: Downstream *pks4* flank. | | | | |
