# Supplementary material for: A novel platform for heterologous gene expression in Trichoderma reesei (Teleomorph Hypocrea jecorina)
Source: Microb Cell Fact. 2014 Mar 6;13:33. doi: 10.1186/1475-2859-13-33 (PMC4015775; doi:10.1186/1475-2859-13-33)
Supplement: Additional file 8: Table S4 — Oligonucleotides used for diagnostic PCR, and Southern blot probes. [file 1475-2859-13-33-S8.docx]

| **Table S4. Oligonucleotides used for diagnostic PCR and Southern blot probes** | | |
| --- | --- | --- |
| Primer name | Sequence (5’-3’) | Target / Usage |
| *tku70*-DT-fw | TGCCTAGGCTCGTCGCGTTT | Confirmation of correct truncation of *tku70* |
| *tku70*-DT-rv | GGGAGCGGATGGTGTTGATAA |  |
| *tku70*-P-fw | GCGATGGACTGTCTCTTCTC | *tku70* |
| *tku70*-P-rv | CGGACCACTCCTCCAAGTAA |  |
| *pyr2*-DT-fw | AGGGCTGGACGTCCACATCG | Confirmation of correct deletion of *pyr2* |
| *pyr2*-DT-rv | CAGGCTTGTGCCAGCCATG |  |
| *ade2*-P-UP-fw | CAACCCATGGCGTAGGGAGG | FL*a*1 |
| *ade2*-P-UP-rv | GCTCAAGTGAGCGACGGCTC |  |
| *ade2*-P-DW-fw | GATCTGACGCTGCGAGCCAG | FL*a*2 |
| *ade2*-P-DW-rv | CGGTGGTTTGAGCGTCTGCG |  |
| exp-IT-UP-fw | GGCCAAAGATCATCCGCAGC | Confirmation of correct upstream insertion of expression fragment |
| exp-IT-UP-rv | CAGTTGCTGAGGGAAGCCGTC |  |
| exp-IT-DW-fw | CCGTAACACCCAATACGCCG | Confirmation of correct downstream insertion of expression fragment |
| exp-IT-DW-rv | CTTGGTTCTAGACGTGGAGGGCC |  |
| *pks4*-DT-fw | GGGAACTAGCTGCCTTGCGTC | Confirmation of correct deletion of *pks4* |
| *pks4*-DT-rv | TAGCTTAACCGCTCACCGTGG |  |
| *ade2*-DT-fw | ACAGGGTAGTCGTGGAACGGAG | Confirmation of correct deletion of *ade2* |
| *ade2*-DT-rv | TTCAACTGCCAGTGTTATACGCC |  |
| lipase-P-fw | ATGAGGAGCTCCCTTGTGCTG | *lip* |
| lipase-P-rv | CTAAAGACATGTCCCAATTAACCCG |  |
| *pyr2*prom-P-fw | GGAGCTGGATGGATGGGCTAAG | *pyr2* promoter |
| *pyr2*prom-P-rv | GCTTTGTGTTGGTTCTTTCCAGGC |  |
| Abbreviations: DT: Deletion test, IT, insertion test, P: Probe, Q: qRT-PCR primer, UP: Upstream, DW: Downstream, FL*a*1: Upstream *ade2* flank, FL*a*2: Downstream *ade2* flank. | | |
